# A Unique Case of Myositis

**DOI:** 10.5041/RMMJ.10481

**Published:** 2022-10-27

**Authors:** Noa Hurvitz, Ariel Kenig, Asa Kessler, Narmine Elkhateeb, Yossef Lerner, Michal Zamir, Fadi Kharouf

**Affiliations:** 1 Department of Medicine, Hadassah Medical Center, Jerusalem, Israel; 2 The Faculty of Medicine, Hebrew University of Jerusalem, Jerusalem, Israel; 3 Department of Neurology, Hadassah Medical Center, Jerusalem, Israel; 4 The Sackler Faculty of Medicine, Tel Aviv University, Tel Aviv, Israel; 5 The Rheumatology Unit, Hadassah Medical Center, Jerusalem, Israel

**Keywords:** ANCA-associated vasculitis, idiopathic inflammatory myopathy, myalgia, myositis, rituximab

## INTRODUCTION

Anti-neutrophil cytoplasmic antibody (ANCA)-associated vasculitis (AAV) is a rare systemic small-vessel disease, with heterogeneous clinical manifestations. While arthralgia and myalgia are common in the disease course, frank myositis is exceedingly rare. Immune-mediated necrotizing myopathy (IMNM) is a subtype of idiopathic inflammatory myopathies (IIMs), characterized by severe myositis. We report herein a case of prominent diffuse myositis with shared features of AAV and IMNM.

## CASE REPORT

A 62-year-old male patient presented with a 2-month history of diffuse myalgia and general weakness. His medical record was notable for past smoking, ischemic heart disease, untreated borderline diabetes mellitus, and stable pulmonary sarcoidosis, for which he received no immunosuppressive therapy. He also reported constitutional symptoms of anorexia, unintentional weight loss (10 kg), and night sweats. Recurrent sinusitis, bilateral toe paresthesia, and numbness in the first four digits of the right hand (dorsal and palmar aspects) were also present. The patient’s muscle aches worsened progressively, eventually limiting his ability to ambulate.

Physical examination revealed a mild symmetric proximal upper and lower limb weakness. Moreover, mild weakness of the wrist extensors and flexors, intrinsic hand muscles, and finger flexors was present. Laboratory tests at admission were remarkable for mild leukocytosis and eosinophilia, marginally elevated creatine phosphokinase, and high inflammatory markers; kidney and liver function tests were normal. Immune serologies revealed elevated rheumatoid factor levels and a positive perinuclear (p−) ANCA test (Table 1). Urinalysis, electrocardiogram, and transthoracic echocardiography were unremarkable. Nerve conduction velocity testing showed minor non-specific changes in the upper limbs, mild right median mononeuropathy (on an unknown background), and mild axonal motor neuropathy in the lower limbs. Electromyography of the quadriceps muscles displayed minor neurogenic changes.

**Table 1:** The Patient’s Admission Blood Tests.

Blood Test (Units)	Normal Range	Results (January 8, 2022)
Creatinine (μmol/L)	62–115	84
AST (U/L)	0–34	42†
ALT (U/L)	10–49	30
ALKP (U/L)	46–116	84
LDH (U/L)	120–246	219
TB (μmol/L)	5–21	10.6
Leukocyte count (10^9^/L)	3.79–10.33	10.7†
Eosinophil count (10^9^/L)	0.03–0.47	0.6†
CRP (mg/dL)	0–0.5	8.7†
ESR (mm/h)	1–20	60†
CPK (U/L)	46–171	313†
Troponin (ng/L)	0–53	27
ANA	0/4	1/4*
C3 (mg/dL)	90–180	165
C4 (mg/dL)	10–40	30.6
IgG (mg/dL)	700–1700	1825
RF (IU/mL)	<14	40.9†
p-ANCA (U/mL)	<5	83†
c-ANCA (U/mL)	<10	2.5

* Considered as negative per local lab standards.

† Abnormal results.

ALKP, alkaline phosphatase; ALT, alanine transaminase; ANA, antinuclear antibody; AST, aspartate aminotransferase; C, complement; c-ANCA, cytoplasmic anti-neutrophil cytoplasmic antibody; CPK, creatine phosphokinase; CRP, C-reactive protein; ESR, erythrocyte sedimentation rate; IgG, immunoglobulin G; LDH, lactate dehydrogenase; p-ANCA, perinuclear anti-neutrophil cytoplasmic antibody; RF, rheumatoid factor; TB, total bilirubin.

**Figure 1:**
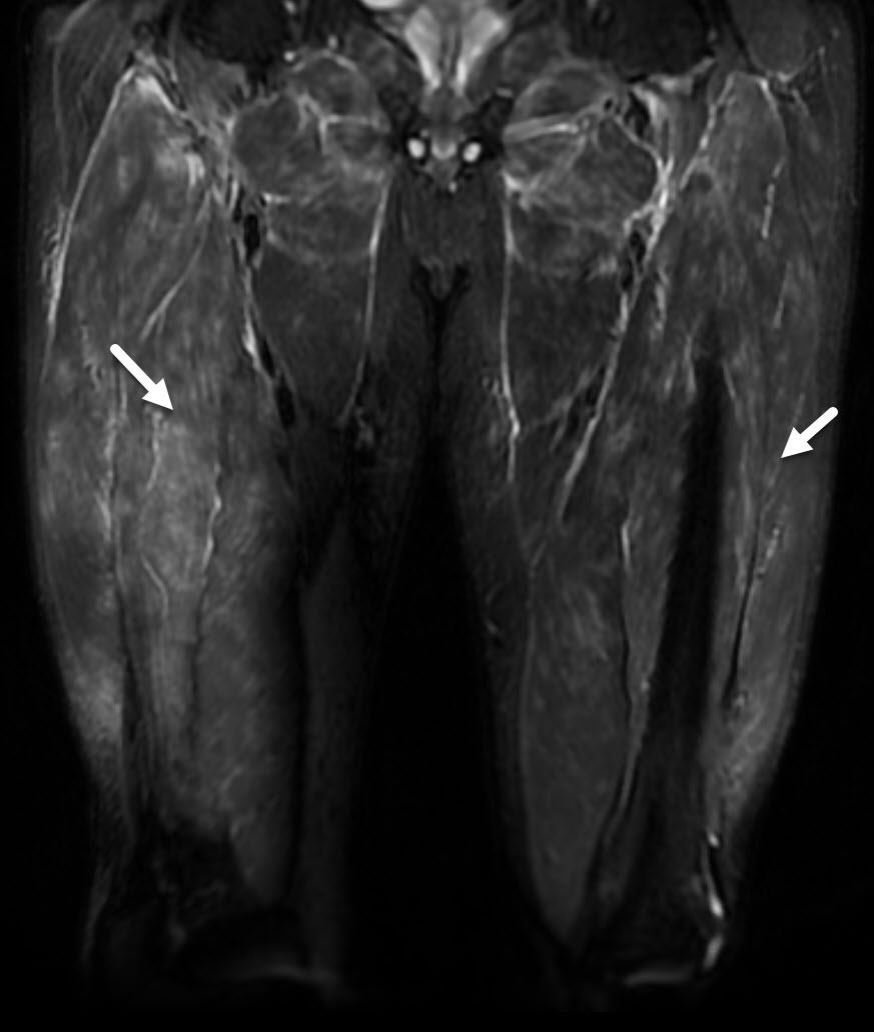
Magnetic Resonance Imaging Scan of the Patient’s Thigh Muscles. Coronal view: short tau inversion sequence (STIR) sequence, showing significant diffuse bilateral muscle edema (arrows).

A total body computed tomography (CT) scan was done, revealing known upper lobe pulmonary emphysema and stable mediastinal lymph nodes, up to 8 mm in size. No evidence of neoplastic or infectious processes was present. Magnetic resonance imaging (MRI) scan of the thighs displayed diffuse myositis in all of the muscle compartments (Figure 1). In light of the clinical and radiologic findings, and in suspicion of an inflammatory myopathy, an open muscle biopsy was obtained. This failed to show prominent signs of muscle inflammation, regeneration, or necrosis; however, deposition of membrane attack complex (MAC) and major histocompatibility complex-I (MHC-I) was detected. Considering the patient’s sensory and motor symptoms in the dorsal and palmar aspects of the right hand, an ischemic central nervous system (CNS) insult was suspected. While a CT angiography scan of the head and neck was unremarkable, a brain MRI scan demonstrated two small ischemic foci in the right lentiform nucleus, compatible with acute stroke.

The patient was diagnosed with AAV, with manifestations of constitutional symptoms, sinusitis, diffuse myositis, peripheral neuropathy, and CNS ischemic lesions. At this stage, the myositis autoantibody panel, which usually takes several weeks to process, returned positive for anti-SRP (signal recognition peptide) antibody, specific for IMNM. Since the patient had features of AAV and IMNM, thorough discussions were carried out regarding his diagnosis. Considering the complexity of the case, the patient was treated with intravenous pulses of methylprednisolone (overall 2.5 g over 3 days), followed by prednisone 60 mg per day. Remarkable clinical recovery ensued within a few days of the initiation of corticosteroids; induction therapy with rituximab was given thereafter.

## DISCUSSION

Frank myositis is an uncommon manifestation of systemic vasculitides. In fact, it has been reported in different vasculitic syndromes, most notably in polyarteritis nodosa, AAV, and rheumatoid vasculitis. Granulomatosis with polyangiitis, microscopic polyangiitis, and eosinophilic granulomatosis with polyangiitis—the three subsets of AAV—have been rarely reported to present with myositis.^1^

When a part of AAV, myositis is usually accompanied by other systemic manifestations of the disease. Muscle enzymes tend to be normal or marginally elevated, and inflammatory markers appear high.^1^ While the imaging findings were traditionally considered indistinguishable from those in IIMs, a recent MRI comparison study suggested that fascial and diffuse subcutaneous fat hyperintensities are more prominent in microscopic polyangiitis than in IIMs.^2^

In a review of 310 muscle biopsies performed in one center, 31 (10%) patients had a final diagnosis of systemic vasculitis. Of these, AAV was present in 22 patients (~71%). In muscle biopsies of patients diagnosed with systemic vasculitis, 66.7% showed signs of small-vessel inflammation, both necrotizing and non-necrotizing; the rest were considered normal. Myalgia and elevated creatine phosphokinase (CPK) were not found to be predictive of muscle biopsy positivity. In this study, the sensitivity of muscle biopsy for detecting vasculitis was overall good (~67%); the specificity was close to 100%.^3^

In another retrospective study of 78 patients with AAV (all of whom underwent muscle biopsy as a part of the diagnostic work-up), 45 (58%) patients had positive biopsies. Anti-myeloperoxidase antibody, female sex, and elevated neutrophil count were predictors of biopsy positivity.^4^

There is scarce evidence in the literature suggesting successful control of myositis in AAV with corticosteroids, methotrexate, azathioprine, mycophenolate mofetil, intravenous immunoglobulin, and cyclophosphamide, among other drugs.^1^

A well-known feature of AAV is CNS involvement. While hypertrophic pachymeningitis is the most frequent CNS presentation, cerebrovascular events, hypophysitis, isolated mass lesions, and seizures may occur. Ischemic infarctions typically present as isolated or multiple lesions affecting the white matter. Peripheral neuropathy may also be a manifestation of AAV.^5^

The muscle biopsy performed on our patient showed no clear necrosis or inflammation, but proved the deposition of MHC-I and MAC. The latter finding signifies an immune-mediated insult.^6^ The absence of clear findings on hematoxylin and eosin staining does not definitely rule out the diagnosis of myositis, since a false-negative rate of more than 20% may be present in muscle biopsies.^7^ The impressive MRI findings further supported our patient’s diagnosis. Fraser et al. found that signal intensity scores on short tau inversion recovery (STIR) were more sensitive in detecting myositis disease activity than was the presence of pathologic changes on muscle biopsy.^8^

Anti-SRP antibody was positive in our patient. Although this autoantibody is considered specific for IMNM, it may also appear in otherwise healthy patients.^9^ It is directed against a ribonucleoprotein complex involved in the regulation of protein translocation across the endoplasmic reticulum. Patients with IMNM tend to have pure myositis with very elevated muscle enzymes; muscle histopathology usually shows scant inflammation and prominent necrosis.^6^

Our patient had features that supported a diagnosis of AAV, including the presence of sinusitis, peripheral neuropathy, mild eosinophilia, only slightly elevated muscle enzymes, and positive p-ANCA. He also had features suggestive of an IIM, including muscle weakness, myalgia, anti-SRP positivity, and the deposition of MAC and MHC-I on biopsy. This combination of findings compatible with AAV and IIM remains extremely rare. Taking into consideration the two diagnoses, therapy with corticosteroids and rituximab was selected.

## CONCLUSION

Our report describes a unique presentation of frank myositis, sinusitis, cerebrovascular insult, and peripheral neuropathy, with evident features of AAV and IMNM. Immunomodulatory therapy led to the patient’s remarkable recovery.

## Abbreviations

AAV: ANCA-associated vasculitis
ANCA: anti-neutrophil cytoplasmic antibodies
CNS: central nervous system
CT: computed tomography
IIMs: idiopathic inflammatory myopathies
IMNM: immune-mediated necrotizing myopathy
MAC: membrane attack complex
MHC-I: major histocompatibility complex-I
MRI: magnetic resonance imaging
p: perinuclear
SRP: signal recognition peptide.
